# *cis*-Jasmone Elicits Aphid-Induced Stress Signalling in Potatoes

**DOI:** 10.1007/s10886-016-0805-9

**Published:** 2017-01-27

**Authors:** Islam S. Sobhy, Christine M. Woodcock, Stephen J. Powers, John C. Caulfield, John A. Pickett, Michael A. Birkett

**Affiliations:** 1 Rothamsted Research, West Common, Harpenden, AL5 2JQ Hertfordshire UK; 2 Department of Plant Protection, Public Service Center of Biological Control (PSCBC), Faculty of Agriculture, Suez Canal University, Ismailia, 41522 Egypt; 3 Department of Microbial & Molecular Systems, KU Leuven, Campus De Nayer, B-2860 Sint-Katelijne-Waver, Leuven, Belgium

**Keywords:** Potato (*Solanum tuberosum* L.), *cis*-Jasmone, Indirect plant defense, *Macrosiphum euphorbiae*, GC-EAG, Olfactometer bioassay

## Abstract

Elicitation of plant defense signaling that results in altered emission of volatile organic compounds (VOCs) offers opportunities for protecting plants against arthropod pests. In this study, we treated potato, *Solanum tuberosum* L., with the plant defense elicitor *cis*-jasmone (CJ), which induces the emission of defense VOCs and thus affects the behavior of herbivores. Using chemical analysis, electrophysiological and behavioral assays with the potato-feeding aphid *Macrosiphum euphorbiae*, we showed that CJ treatment substantially increased the emission of defense VOCs from potatoes compared to no treatment. Coupled GC-electroantennogram (GC-EAG) recordings from the antennae of *M. euphorbiae* showed robust responses to 14 compounds present in induced VOCs, suggesting their behavioral role in potato/aphid interactions. Plants treated with CJ and then challenged with *M. euphorbiae* were most repellent to alate *M. euphorbiae*. Principal component analysis (PCA) of VOC collections suggested that (*E*)-2-hexenal, (*E*,*E*)-4,8,12-trimethyl-1,3,7,11-tridecatetraene (TMTT), (*E*)-β-farnesene, (*E*)-4,8-dimethyl-1,3,7-nonatriene (DMNT), methyl salicylate (MeSA), CJ, and methyl benzoate (MeBA) were the main VOCs contributing to aphid behavioral responses, and that production of TMTT, (*E*)-β-farnesene, CJ, and DMNT correlated most strongly with aphid repellency. Our findings confirm that CJ can enhance potato defense against aphids by inducing production of VOCs involved in aphid-induced signalling.

**Electronic supplementary material:**

The online version of this article (doi:10.1007/s10886-016-0805-9) contains supplementary material, which is available to authorized users.

## Introduction

Plants have evolved sophisticated mechanisms against herbivore attack, including production of complex mixtures of volatile organic compounds (VOCs) that directly deter herbivores (Bruce and Pickett 2011; Mithöfer and Boland 2012). This phenomenon has raised the prospect of manipulating the emission of VOCs to enhance crop protection (Heil 2014; Turlings and Ton 2006). Such qualitative or quantitative manipulation of VOCs is achievable by boosting signal transduction pathways leading to volatile emission (Thaler et al. 2012), and elicitors are known to increase and/or decrease VOC emission by acting on these signaling pathways (Smith et al. 2009).

*cis*-Jasmone (CJ) is a plant-derived natural product that is biosynthesized *via* isomerization of *cis*-oxophytodienoic acid (*cis*-OPDA) to *iso*-oxophytodienoic acid (*iso*-OPDA), followed by oxidative side-chain cleavage (Dabrowska and Boland 2007). Plants release CJ upon herbivory (Birkett et al. 2000; Loughrin et al. 1995; Röse and Tumlinson 2004), application of insect saliva (Lou and Baldwin 2003; Röse and Tumlinson 2005; Sobhy et al. 2015), treatment with jasmonic acid (JA) (Heil 2004), or inoculation with nitrogen-fixing rhizobia (Ballhorn et al. 2013). Moreover, CJ is also constitutively released by many flowers and sometimes by leaves as an attractant for pollinators (Tanaka et al. 2009), or as a chemical cue for host location by insect flower herbivores (El-Sayed et al. 2009). Given the structural similarity between CJ and JA, and the production of CJ as a stress signal, the ability of CJ to elicit plant defense has been studied in crop plants, e.g. cereals (Bruce et al. 2003a; Moraes et al. 2008; Oluwafemi et al. 2013), soybean (Moraes et al. 2009) and cotton (Hegde et al. 2012). *cis*-Jasmone treatment of crop plants not only induces direct defense against herbivores, but also induces indirect defense by releasing VOCs that attract natural enemies (Birkett et al. 2000; Bruce et al. 2008; Moraes et al. 2009; Vieira et al. 2013). However, it should be noted that, in addition to the divergent synthesis of CJ from that of JA, CJ also signals differently to JA (Matthes et al. 2010, 2011).

Recently, potatoes, *Solanum tuberosum* L. (Solanaceae), have become regarded more as a staple crop after a dramatic increase in cereal prices (FAO 2012). An increase in consumption of potatoes clearly requires an increase in the yield per unit cultivated area (FAO 2009). However, insect infestation is a major constraint reducing worldwide potato production. Virus vectors such as aphids (Homoptera: Aphididae) (Saguez et al. 2013) are a notable pest. The potato aphid, *Macrosiphum euphorbiae* Thomas, is a polyphagous species that feeds on 200 plant species belonging to 20 different families, including several *Solanum* species (Le Roux et al. 2010). *M. euphorbiae* is often reported as the second most important virus vector after the peach-potato aphid, *Myzus persicae* Sulzer, as it can transmit over 45 plant viruses (Fuentes et al. 1996). Currently, aphids are controlled worldwide through extensive use of insecticides, but future use is under scrutiny due to mounting problems of insecticide resistance (Radcliffe et al. 2009). Due to likely reductions in insecticide availability and registration, alternative control strategies are urgently sought (Van Doorn and de Vos 2013). Biological control is an alternative but is insufficiently effective, and is applied only on a relatively small acreage (van Lenteren 2012). It has been proposed that enhancing the release of defense VOCs in crop plants may improve biological control (Heil 2014; Sobhy et al. 2014; Turlings and Ton 2006). Plant elicitors (or activators) such as CJ could, therefore, be used to achieve this for potato crops. Given our previous promising findings on the use of CJ to elicit defense against aphid pests on some crop species, here we tested, for the first time, the hypothesis that CJ elicits aphid-induced signalling in potatoes, by targeting the pest aphid, *M. euphorbiae*.

## Methods and Materials

### Plant Material and *cis*-Jasmone Application

Potato plants, *S. tuberosum*. cv. Désirée, were grown in a glasshouse at 25 ± 2 °C under a 16L: 8D h photoperiod. In all experiments, 3-wk-old potato plants (30–45 cm height stage) were used. Potato plants were sprayed with an aqueous emulsion of *cis*-jasmone (CJ) (90%; Avocado, Lancaster, UK). Spray treatments were carried out using a hydraulic nozzle (Brown 015-F110) mounted on a variable speed spray track at 1 ms^−1^ (Pressure 3.0 bar; height above plants 35 cm; swath width 0.5 m). For CJ treatment, 25 µl of CJ and 100 μl of Ethylan BV in 100 ml of deionized water were applied (Dewhirst et al. 2012), whereas 100 μl of Ethylan BV in 100 ml of deionized water were used for the control treatment (SUR) with an application rate of 200 l h^−1^. Ethylan BV was used to emulsify CJ in water and lower the interfacial tension between the liquid and the plant’s epidermis to encourage uptake (Hazen 2000). Sprayed plants were kept in a glasshouse for 1 d and then subjected on the second day to either immediate headspace collection, or infestation of *M. euphorbiae* and then headspace collection.

### Insects

Potato aphids, *M. euphorbiae*, as apterous virginoparae, were collected from infested potato plots in a single field at Rothamsted Research (51.8096° N, 0.3563° W). Laboratory colonies then were established from parthenogenetic individuals that were maintained on *S. tuberosum* cv. Désirée in ventilated polypropylene breeding cages (30 × 30 × 30 cm, Bugdorm 1; Watkins & Doncaster, Kent, UK) in a controlled environment room (20 ± 1 °C, 60 ± 10% RH, 16L: 8D h photoperiod), which ensured continuous asexual reproduction. Apterous aphids were used in volatile collection experiments, whereas migrating alate morphs, obtained by overcrowding, which is widely known as a main factor to influence wing production in aphids (Mehrparvar et al. 2013), were used in olfactometer bioassays and electrophysiology experiments (Dewhirst et al. 2012).

### Plant Treatments

Potato plants were allocated randomly to one of the following treatments: (a) blank control (INTACT): either untreated or un-infested plants; (b) control formulation (SUR): 0.1% non-ionic surfactant Ethylan BV in water; (c) *cis*-jasmone formulation (CJ): 0.1% aqueous EBV plus CJ; (d) aphid-infested plants (ME): plants were challenged with 100 apterous individuals; (e) CJ and then infestation with aphid individuals (CJME): plants were challenged with 100 apterous individuals 24 hr after *cis*-jasmone treatment. This gave a control (a) plus two-by-two factorial treatment structure (b-e) for the presence/absence of aphids by presence/absence of CJ. Prior to being used in experiments, sprayed plants with CJ were kept in separate glasshouse compartments (20 ± 1 °C, 25–40% RH, 16L: 8D h photoperiod) in order to minimize unwanted induction of plant defense by plant/plant volatile interactions between treatments.

### Volatile Organic Compound (VOC) Collection

Dynamic headspace collection was carried out following standard procedures (Webster et al. 2008). For each collection, a compound leaf with 5–7 leaflets was enclosed in a glass vessel (22 cm high × 10 cm internal diameter), which was open at the bottom and with two collection ports at the top (one for inlet of air and the other for outlet). The bottom was closed without pressure around the plant stem, using two semicircular aluminum plates with a hole in the center to accommodate the stem. The plates were clipped to the base of the glass vessel without constricting the plant. Air, purified by passing through an activated charcoal filter (BDH, 10–14 mesh, 50 g), was pushed into the vessel through the inlet port at 700 ml min^−1^ (flow rate controlled by a needle valve and measured by a flow meter). Air was pulled out at 500 ml min^−1^ through Porapak Q 50/80 (50 mg, Supelco, Bellefonte, PA, USA) held by two plugs of silanized glass wool in a 5 mm diameter glass tube (Alltech Associates, Carnforth, Lancashire, UK). All connections were made with polytetrafluoroethylene (PTFE) tubing (Alltech Associates) with brass ferrules and fittings (North London Valve, London, UK) and sealed with PTFE tape. Glassware, metal plates and other equipment were washed with Teepol detergent (Teepol, Kent, UK) in an aqueous solution, acetone and distilled water, and then baked overnight at 180 °C. Porapak Q tubes were conditioned before use by washing with redistilled diethyl ether (4 ml) and heated to 132 °C under a stream of purified nitrogen and kept for 2 hr. Diethyl ether was purchased from Sigma Aldrich and distilled prior to use. VOC extracts required for olfactometry, GC, GC-EAG, and GC–MS analysis were collected for 120 hr (5 successive days) in 24 hr periods. After each collection period, VOCs in Porapak Q tubes were eluted with freshly redistilled diethyl ether (750 μl). The samples were concentrated under a stream of nitrogen to ∼50 μl and stored at -20 °C until required for analysis. Separate VOC extracts required for quantification studies were obtained from each of three independent plants (biological replicates) per treatment. Using new, i.e. different, potato plants, another collection set was obtained from three independent plants per treatment for behavioral and electrophysiological assays. In all experiments, VOCs were collected from five treatments: (i) INTACT = untreated plants; (ii) SUR = EBV treated plants; (iii) CJ = CJ treated plants; (iv) ME = aphid infested plants; (v) CJME = CJ treated plants then infested with aphids. For the CJME treatment, plants were challenged with aphids 24 hr after CJ treatment. For ME and CJME treatments, intact plants and plants sprayed with CJ were challenged with 100 apterous individuals that were collected into small glass vials. In all experiments, air entrainment was carried out under controlled conditions (20 °C, 60% RH, 16L: 8D h photoperiod).

### Chemical Analysis

Volatile organic compound (VOC) extracts were analyzed by gas chromatography (GC) using an Agilent 6890 GC equipped with a cool on-column injector, flame ionization detector (FID) and a nonpolar HP-1 capillary column (50 m × 0.32 mm inner diam., film thickness 0.5 μm; J & W Scientific). The GC oven temperature was maintained at 30 °C for 1 min after sample injection and then raised by 5 °C min^−1^ to 150 °C, then by 10 °C min^−1^ to 230 °C. The carrier gas was hydrogen (10 psi), and 4 μl of each eluted sample were injected into the injector port of the GC instrument. HP Chemstation software was used for data analysis.

Coupled gas chromatography-mass spectrometry (GC-MS) was performed on an Agilent MSD 5972 and Agilent 5890 GC (fitted with a non-polar HP1 column 50 m length × 0.32 mm inner diam. × 0.52 μm film thickness, J & W Scientific). Sample injection was *via* a cool on-column injector port with helium as the carrier gas, and ionization was by electron impact (70eV, source temperature 220 °C). The GC oven temperature was maintained at 40 °C for 1 min and then programmed at 5 °C min^−1^ to 250 °C, run time 60 min.

Tentative identifications were made by comparison of spectra with mass spectral databases (NIST, 2005). Peak enhancement by co-injection with authentic standards was undertaken to confirm tentative identification (Pickett 1990). Chemicals (*>*95% pure) were obtained from commercial sources (Sigma-Aldrich, Gillingham, Kent, UK; Botanix Ltd., Paddock Wood, Kent, UK), apart from (*E*)-β-farnesene, which was synthesized in one step from farnesyl chloride (Kang et al. 1987) and (*E*)-4,8-dimethyl-1,3,7-nonatriene (DMNT) and (*E*,*E*)-4,8,12-trimethyl-1,3,7,11-tridecatetraene (TMTT), which were synthesized from geraniol and (*E,E*)-farnesol, respectively, by oxidation to their corresponding aldehydes, followed by Wittig methylenation (Leopold 1986). The purities of synthesized (*E*)-β-farnesene, DMNT and TMTT were >98%. The quantities of compounds present in VOC extracts were determined according to the weight of sampled plant material, and the duration of the entrainment period.

### Coupled GC-Electrophysiology

Electroantennogram (EAG) recordings were made with alate *M. euphorbiae* using Ag-AgCl glass electrodes filled with a saline solution but without glucose (Maddrell 1969). The head of the aphid was removed and placed within the indifferent electrode. To ensure a good contact, the ends of the antennae, after removing the tips, were inserted into the recording electrode. The signals were passed through a high impedance amplifier (UN-06; Syntech, Hilversum, The Netherlands) and analyzed with a customized software package (EAD version 2.3; Syntech, Hilversum, The Netherlands). Coupled gas chromatography-electroantennography (GC-EAG), in which the effluent from the GC column is simultaneously directed to the antennal preparation and the GC detector, has been described previously (Wadhams 1990). The effluent from the transfer line to the antenna was delivered into a purified airstream (1 L min ^−1^) flowing continuously over the preparation. Separation of the VOCs was achieved on an AI 93 GC equipped with a cool on-column injector, an FID and an HP-1 capillary column (50 m × 0.32 mm inner diam.). The oven temperature was maintained at 30 °C for 2 min, and then programmed to increase at 15 °C min^−1^ to 250 °C, run time 60 min. The carrier gas was helium (16.6 psi). The outputs from the EAG amplifier and the FID were monitored simultaneously and analyzed using the Syntech software package (Syntech, Hilversum, The Netherlands). FID peaks were assumed to be EAG active if they elicited responses on three or more preparations.

### Olfactometry

The behavioral responses of alate *M. euphorbiae* to potato VOCs were investigated using a Perspex 4-arm olfactometer in a controlled environment room (22 ± 2 °C, 40% RH) fitted with an extractor fan. The central area at the top of the olfactometer contained a hole into which a single alate *M. euphorbiae* was introduced, and which was connected to a low pressure air pump. Air was removed from the center of the olfactometer by a vacuum pump, buffered by a 2 L jar and adjusted with a flow meter to 400 ml min^−1^. Polytetrafluoroethylene (PTFE) tape was used to ensure airtight seals between the olfactometer and the Teflon tubing. All five holes were covered with a layer of muslin to prevent access by an aphid during the bioassays. To eliminate any visual cues, the olfactometer was placed in a black cage (60 × 60 × 76 cm) comprising a steel frame covered with black cardboard paper with an observation opening at the front. Uniform illumination was provided by two fluorescent light tubes (70 W Luminux) positioned approximately 45 cm above the olfactometer. The olfactometer arena was split into five areas: four areas by each arm [(one or two treatment arm(s) and three, or two, control arms)] and a central area (Webster et al. 2010). Adult winged aphids were collected from rearing cages in a separate insectary room and starved for 2 hr before each trial. Each aphid was exposed to a test sample for 16 min, and after every 2 min the position of the olfactometer was rotated clockwise by 90° to eliminate bias. The number of entries made by *M. euphorbiae* into the different arms of the olfactometer and time spent in them were recorded using a software program (OLFA, F. Nazzi, Udine, Italy). Two randomized block experiments were done. A first study was conducted to test VOC extracts collected from each of two periods, 24–48 hr and 72–96 hr, separately i.e., (i) INTACT_48h or 96h_ potato plants vs. a solvent control (diethyl ether), (ii) CJ_48h or 96h_ sprayed potato plants vs. a solvent control (diethyl ether), (iii) ME_48h or 96h_ potato plants infested with aphids vs. a solvent control (diethyl ether), and (iv) CJME_48h or 96h_ CJ- sprayed potato plants then infested with aphids vs. a solvent control. Ten replicates were done for each comparison. For each experiment, filter paper (185 mm diameter; Whatman Filter Paper, Maidstone, UK) strips were each treated with an aliquot (10 μl) of the test solution, applied using a micropipette (Drummond ‘microcaps’; Drummond Scientific Co., USA), and the solvent was allowed to evaporate for 30 sec. One arm was assigned to the collected VOCs from treated plants, whereas the other three control vessels were treated similarly with the same volume of solvent (diethyl ether) on filter paper strips (solvent control). Furthermore, a second olfactometer experiment was conducted to test directly four comparisons: i.e., (i) CJ_48h_ sprayed potato plants vs. INTACT_48h_ potato plants, (ii) ME_48h_ potato plants vs. INTACT_48h_ potato plants, (iii) CJME_48h_ potato plants vs. INTACT_48h_ potato plants, and (iv) CJME_48h_ potato plants vs. ME_48h_ potato plants. In this set-up, each treatment was assigned to one arm, and the other two arms were assigned to solvent control (diethyl ether). Likewise, a similar set-up was performed to test VOCs collected after 72–96 hr air entrainment. Ten replicates were done for each comparison. All bioassays were performed between 9:00 A.M. and 5:00 P.M.

### Statistical Analysis

Two-way ANOVA was used to evaluate whether CJ and aphids, as main factors, had any interaction effects on the total amount of VOCs that were trapped at each of the collection points (i.e.,: 0–24, 24–48, 48–72, 72–96 and 96–120 hr). Prior to this analysis, data at each collection point were examined for conformation to a Normal distribution using the Shapiro–Wilk test, and homogeneity of variances was tested by the Levene test. The results of these tests confirmed that the data were consistent with the assumptions for valid application of ANOVA. To examine the impact of each independent treatment on the total emitted VOCs, one-way ANOVA was also performed on the data from each collection point separately, followed by application of the Student-Newman-Keuls method to separate the five means.

Out of 29 VOCs that were tentatively identified from the VOC blends of potato plants, only 14 compounds were shown by GC-EAG to be electrophysiologically active. To elucidate how each of these 14 EAG-active VOCs contributed to explaining the variation in the blends obtained from different potato treatments, a multivariate statistical technique, principal component analysis (PCA), was applied, given that each treatment has three independent biological replicates and, incorporating each compound as a variable according to Rencher (2002). Taking into account the patterns of correlations between compounds, the linear combinations of them (i.e., the principal components, PCs) also provided a visual representation of the treatments (Hare 2011). We used two types of output: a matrix of ‘scores’, which provides the location of each sample on each PC, and a matrix of ‘loadings’ which indicates the strength of correlation between individual VOCs and each PC (Babikova et al. 2013). PCA was carried out using PAST - Paleontological Statistics, Version 2.17 (Hammer et al. 2001).

Given the significant differences for both factors (CJ and Aphid) on total emitted VOCs that were found at 24–48 and 72–96 hr collection points (Table 1), we conducted a second two-way ANOVA to test whether there were any interactions between these factors for the 14 EAG active VOCs (that were identical with the co-injection of authentic samples), and means were thereafter separated using the Student-Newman-Keuls method. Such a (2 × 2) factorial model easily allows for assessing additive vs. synergistic interactions of the treatment main factors (Kutner et al. 2005). However, to test the effect of each independent treatment, one way ANOVAs were performed, and then the Student-Newman-Keuls method was used to compare treatment means at 24–48 and 72–96 hr collection points. The results of Shapiro-Wilks and Levene tests also confirmed that these data were consistent with the assumptions for valid application of ANOVA.

**Table 1:** Results of two-way ANOVA testing the effects of the different treatment factors (*cis*-Jasmone and Aphid) and their interaction (*cis*-Jasmone x Aphid) on the total emitted potato VOCs at each of the five 24 hr collection points

Sources	0–24 hr VOCs			24–48 hr VOCs			48–72 hr VOCs			72–96 hr VOCs			96–120 hr VOCs		
	*df*	*F*	*P*	*df*	*F*	*P*	*df*	*F*	*P*	*df*	*F*	*P*	*df*	*F*	*P*
*cis*-Jasmone	1	0.103	0.754	1	14.423	**0.003**	1	0.727	0.412	1	27.954	**<0.001**	1	20.617	**<0.001**
Aphid	1	0.520	0.486	1	32.510	**<0.001**	1	51.680	**<0.001**	1	61.866	**<0.001**	1	1.520	0.243
*cis*-Jasmone x Aphid	1	0.556	0.471	1	0.189	0.672	1	0.0869	0.774	1	0.129	0.727	1	0.325	0.580
Residual	11			11			11			11			11		

*P*-values in bold indicate significant differences between treatments (*P* < 0.05)

The behavioral response of *M. euphorbiae* was tested in two ways. First, for experiments with one treated arm vs. three solvent control treatments, data were analyzed by a paired *t*-test, under the hypothesis that aphids should be repelled by the emitted VOCs under certain conditions (Dewhirst and Pickett 2009; Hegde et al. 2011, 2012). In this analysis, the time spent and entries by aphids into treated and control arms of the four-arm olfactometer were compared. In experiments where the response in two treated arms vs. two arms of solvent control was compared, data were examined for a Normal distribution using the Shapiro-Wilk test before analysis. When a Shapiro-Wilk test indicated that data were distributed as Normal, they were analyzed by parametric analysis of variance (ANOVA) followed by the Student-Newman-Keuls method for separation of means. When data were not distributed as Normal, a nonparametric Kruskal-Wallis (H test) was used followed by Dunn’s method for separation of means. All analyses were performed using SPSS (SPSS Inc., Chicago, IL, USA).

## Results

### VOC Analyses

**Fig. 1:**
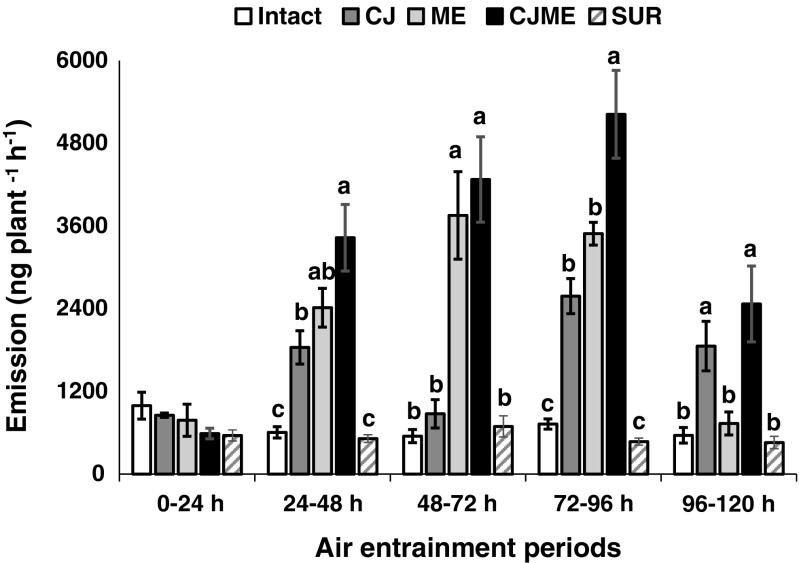
Total amount (mean nanograms plant^−1^ h^−1^ ± SE) of volatile organic compounds (VOCs) emitted from potato (*Solanum tuberosum*) plants following different treatments: INTACT plants = neither *cis*-jasmone (CJ) treatment nor *Macrosiphum euphorbiae*–infested, CJ = CJ treatment, ME = *M. euphorbiae*–infested, CJME = CJ treatment and then infestation with *M. euphorbiae*, SUR = surfactant treatment. For aphid treatments, each plant was infested with 100 apterous individuals. For each collection period, different letters indicate statistically significant differences between treatments (*P* < 0.05, Student-Newman-Keuls method)

Gas chromatography (GC) and coupled GC-mass spectrometry (GC-MS) analyses of volatile collections from potato plants revealed the presence of 29 detectable VOCs under different treatments (i.e. INTACT plants = neither cis-jasmone (CJ) nor *Macrosiphum euphorbiae*–infested; CJ = CJ treatment; ME = *M. euphorbiae*–infested; CJME = CJ treatment then infestation afterwards with *M. euphorbiae*; SUR = surfactant treatment). Generally, excluding the first VOC collection period (0–24 hr), the volatile emission of INTACT and SUR plants was at least three-fold less than other treatments (i.e. CJ, ME and CJME). Statistical analysis revealed that the total emitted VOCs from CJ, ME and CJME plants were higher (2–4 fold) compared to SUR or INTACT plants (one-way ANOVA: 48 h: *F*_4,14_ = 13.35, *P* < 0.001; 72 h: *F*_4,14_ = 12.88, *P *< 0.001; 96 h: *F*_4,14_ = 25.73, *P* < 0.001; 120 h: *F*_4,14_ = 5.57, *P* = 0.013). The total VOC emissions from potato plants collected during the 96–120 hr period for all treatments were notably decreased (2 fold) compared to the 72–96 hr period (Fig. 1).

Two-way ANOVA analysis of the total emitted VOCs showed that no interaction occurred between the main factors (CJ and Aphid) at any point of VOC collection (Table 1). However, one or both factors had a statistically significant impact on the total emitted VOCs at all collection points except for the first period (0–24 hr). Indeed, both factors had a significant impact on the released VOCs at the collection points of 24–48 hr (CJ: *F*_1,14_ = 14.42, *P* = 0.003; Aphid: *F*_1,14_ = 32.51, *P* < 0.001) and 72–96 hr (CJ: *F*_1,14_ = 27.95, *P* < 0.001; Aphid: *F*_1,14_ = 61.86, *P* < 0.001), which supported the proposal to analyze the EAG-active VOCs at these collection points. Statistical analysis of the individual VOCs that were collected during 24–48 hr and 72–96 hr, using two-way ANOVA analysis, revealed a significant interaction between CJ treatment and aphid herbivory. Certain compounds, such as DMNT and (*E*)-β-farnesene, were significantly (*P* < 0.05, F-tests) affected by both factors in a synergistic manner during the 24–48 hr period. Such synergetic interaction between aphid herbivory and CJ treatment was notably enhanced with time, as further VOCs, i.e. 6-methyl-5-hepten-2-one (MHO), decanal, indole and CJ, were significantly (*P* < 0.05, F-tests) affected in this way at 72–96 hr but not at 24–48 hr (Table 2).

**Table 2:** Results of two-way ANOVA testing the effects of the different treatment factors (*cis*-Jasmone and Aphid) and their interaction (*cis*-Jasmone x Aphid) on potato VOC emission at 24–48 and 72–96 hr collection points

Plant volatile	24–48 hr VOCs						72–96 hr VOCs					
	*cis*-Jasmone		Aphid		*cis*-Jasmone x Aphid		*cis*-Jasmone		Aphid		*cis*-Jasmone x Aphid	
	*F*	*P*	*F*	*P*	*F*	*P*	*F*	*P*	*F*	*P*	*F*	*p*
(*E*)-2-Hexenal	4.14	0.067	11.98	**0.005**	0.02	0.890	0.12	0.731	15.86	**0.002**	0.07	0.794
α-Pinene	0.81	0.388	1.76	0.212	0.13	0.727	0.20	0.661	3.68	0.081	0.07	0.795
MHO	2.59	0.136	24.77	**<0.001**	2.91	0.117	14.02	**0.003**	16.98	**0.002**	38.65	**<0.001**
MeBA	1.03	0.331	8.19	**0.015**	2.79	0.123	6.35	**0.028**	9.87	**0.009**	1.26	0.286
Nonanal	0.001	0.981	2.737	0.126	0.08	0.775	0.04	0.850	19.36	**0.001**	0.09	0.762
DMNT	1.29	0.281	7.48	**0.019**	7.18	**0.021**	30.34	**<0.001**	16.52	**0.002**	9.36	**0.011**
(*Z*)-3-Hexen-1-yl butyrate	0.003	0.951	6.37	**0.028**	0.01	0.915	0.78	0.395	3.47	0.089	1.18	0.300
MeSA	2.92	0.116	5.88	**0.034**	1.56	0.238	8.53	**0.014**	18.16	**0.001**	0.09	0.761
Decanal	0.008	0.929	0.42	0.533	4.74	0.052	3.33	0.095	2.07	0.178	10.58	**0.008**
Indole	2.17	0.169	0.003	0.953	1.21	0.295	1.02	0.334	0.21	0.655	5.72	**0.039**
*cis*-Jasmone	14.81	**0.003**	27.99	**<0.001**	4.39	0.060	57.09	**<0.001**	179.50	**<0.001**	41.17	**<0.001**
α-Copaene	1.17	0.301	0.078	0.785	0.52	0.487	1.91	0.194	0.21	0.657	0.69	0.422
(*E*)-β-Farnesene	1.11	0.315	4.98	**0.047**	7.844	**0.017**	16.03	**0.002**	5.92	**0.033**	2.08	0.177
TMTT	11.82	**0.006**	11.47	**0.006**	0.22	0.647	8.24	**0.015**	16.76	**0.002**	0.62	0.449

All *F*-tests were on 1 (for factors) and 11 (for residual) degree of freedom (*df*)

The mean amount (±SE) of individual VOCs released by potato plants under each treatment at the collection points of 24–48 hr and 72–96 hr are shown in Table 3. Here, the impact of each independent treatment was tested using one way analysis of variance (ANOVA). Potato plants released higher amounts (≥ 2 fold) of particular VOCs when they were treated with CJ and then challenged with aphids (CJME) compared to the other treatments (Table 3). Furthermore, CJ and/or aphid (ME) treatments significantly (*P* < 0.05, Student-Newman-Keuls) affected the emission of many VOCs compared to control (SUR), indicating that each treatment alone could also induce the VOC emission, but aphid herbivory induced the VOC emission more than CJ treatment.

**Table 3:** Mean amount of volatile organic compounds (VOCs) (in ng; mean ± SE) emitted by potato plants under different treatments (i.e.,: INTACT plants = neither *cis*-jasmone nor aphid treatment; CJ = *cis*-jasmone treatment; ME = *Macrosiphum euphorbiae*–infested; CJME = *cis*-jasmone treatment then infestation afterwards with *M. euphorbiae*; SUR = surfactant treatment). Air entrainment was conducted for 120 hr in five periods of 24 hr. Data shown here are for the 24–48 hr and 72–96 hr VOC collection points. Results (*P*-values) of one-way ANOVA are given

Compounds		Potato VOCs collected during 24–48 hr air entrainment						Potato VOCs collected during 72–96 hr air entrainment					
		INTACT	CJ	ME	CJME	SUR	*P* value	INTACT	CJ	ME	CJME	SUR	*P* value
1	(*E*)-2-Hexenal	n.d.^c^	37.9 ± 1.9^ab^	60.7 ± 9.3^ab^	90.8 ± 30.8^a^	6.7 ± 3.6 ^b^	**0.026**	3.1 ± 0.5^b^	8.1 ± 0.9^b^	176.3 ± 17.2^a^	205.1 ± 93.3^a^	5.2 ± 2.2^b^	**0.045**
2	*α*-Pinene	7.8 ± 1.4	23.9 ± 5.1	27.3 ± 11.3	31.7 ± 8.8	19.7 ± 3.9	0.403	23.6 ± 2.8	39.17 ± 10.8	99.7 ± 37.2	129.3 ± 72.9	39.1 ± 16.7	0.460
3	MHO	12.9 ± 3.1 ^b^	19.1 ± 9.4 ^b^	174.8 ± 35.5^a^	96.5 ± 30.9^ab^	20.7 ± 3.6^b^	**0.006**	15.9 ± 8.4^c^	67.5 ± 16.9^b^	249.9 ± 42.3^a^	18.9 ± 9.1^bc^	4.5 ± 1.5^c^	**˂0.001**
4	MeBA	6.7 ± 3.2	12.6 ± 5.1	33.3 ± 3.9	18.9 ± 5.5	11.6 ± 5.7	0.069	28.9 ± 7.4^c^	106.8 ± 33.8^b^	134.4 ± 34.8^b^	296.3 ± 75.5^a^	60.3 ± 2.6^bc^	**0.026**
5	Nonanal	37.7 ± 10.4	89.7 ± 19.1	148.8 ± 33.9	137.6 ± 36.1	122.9 ± 32.3	0.229	60.6 ± 16.3^c^	94.5 ± 19.7^b^	223.1 ± 29.2^b^	238.5 ± 19.1^a^	135.7 ± 33.3^b^	**0.006**
6	DMNT	4.4 ± 1.9 ^b^	309.1 ± 38.9^a^	439.8 ± 131.8^a^	313.5 ± 90.7^a^	n.d. ^b^	**0.019**	33.3 ± 6.9^b^	494.3 ± 50.1^a^	414.7 ± 33.2^a^	548.6 ± 92.2^a^	25.1 ± 10.3^b^	**˂0.001**
7	(*Z*)-3-Hexen-1-yl butyrate	4.9 ± 2.3	5.4 ± 1.7	77.4 ± 49.2	78.7 ± 27.8	15.4 ± 5.4	0.171	10.7 ± 6.2	5.5 ± 1.5	13.4 ± 6.8	29.5 ± 13.3	3.6 ± 1.7	0.757
8	MeSA	1.2 ± 0.9	5.1 ± 2.9	12.1 ± 5.9	41.2 ± 18.9	n.d.	0.302	11.6 ± 1.9^c^	63.8 ± 14.7^b^	90.3 ± 23.7^ab^	154.3 ± 29.1^a^	12.7 ± 3.5^c^	**0.005**
9	Decanal	112.4 ± 29.7	75.9 ± 40.1	44.2 ± 21.3	142.3 ± 8.3	220.1 ± 53.9	0.103	129.7 ± 18.6^a^	53.9 ± 15.9^b^	44.3 ± 10.6^b^	171.2 ± 23.8^a^	49.6 ± 16.4^b^	**0.006**
10	Indole	173.5 ± 55.6^a^	n.d.^c^	57.3 ± 18.1 ^b^	41.9 ± 11.6 ^b^	34.2 ± 5.9 ^b^	**0.038**	62.5 ± 6.2	145.5 ± 26.3	129.6 ± 24.1	91.5 ± 42.5	36.1 ± 14.8	0.161
11	*cis*-Jasmone	n.d.^c^	26.6 ± 3.4^b^	48.5 ± 16.6^b^	138.9 ± 26.1^a^	n.d.^c^	**0.013**	4.7 ± 1.7^c^	15.9 ± 5.6^c^	70.3 ± 16.1^b^	200.3 ± 8.3^a^	6.1 ± 0.9^c^	**˂0.001**
12	*α*-Copaene	175.7 ± 78.7	5.7 ± 1.9	46.5 ± 17.6	28.1 ± 11.9	18.9 ± 7.5	0.062	118.9 ± 15.4^a^	78.51 ± 26.7^a^	22.7 ± 6.8^b^	89.9 ± 26.1^a^	4.7 ± 1.9^b^	**0.024**
13	(*E*)-*β*-Farnesene	n.d.^c^	18.3 ± 2.2^ab^	23.1 ± 5.9^a^	15.9 ± 4.7^a^	4.9 ± 2.1^bc^	**0.022**	2.7 ± 1.2^c^	16.7 ± 0.8^b^	8.3 ± 5.5^bc^	37.8 ± 9.4^a^	3.1 ± 1.5^c^	**0.011**
14	TMTT	n.d.^b^	593.1 ± 189.1^a^	585.3 ± 176.9^a^	1035.3 ± 171.3^a^	n.d.^b^	**0.007**	18.9 ± 2.9^b^	541.7 ± 121.7^a^	714.8 ± 72.5^a^	1023.5 ± 262.1^a^	n.d.^b^	**0.005**

The compounds are ordered in accordance with their increasing GC retention time. Different letters indicate significant differences between treatments (*P* < 0.05, Student-Newman-Keuls method) when the one-way ANOVA result was significant (*P* < 0.05, *F*-test). For aphid infested plants (ME or CJME), potato plants were challenged with 100 apterous individuals of *M. euphorbiae*

*P*-values in bold indicate significant differences between treatments (*P* < 0.05)

### Electrophysiology

**Fig. 2:**
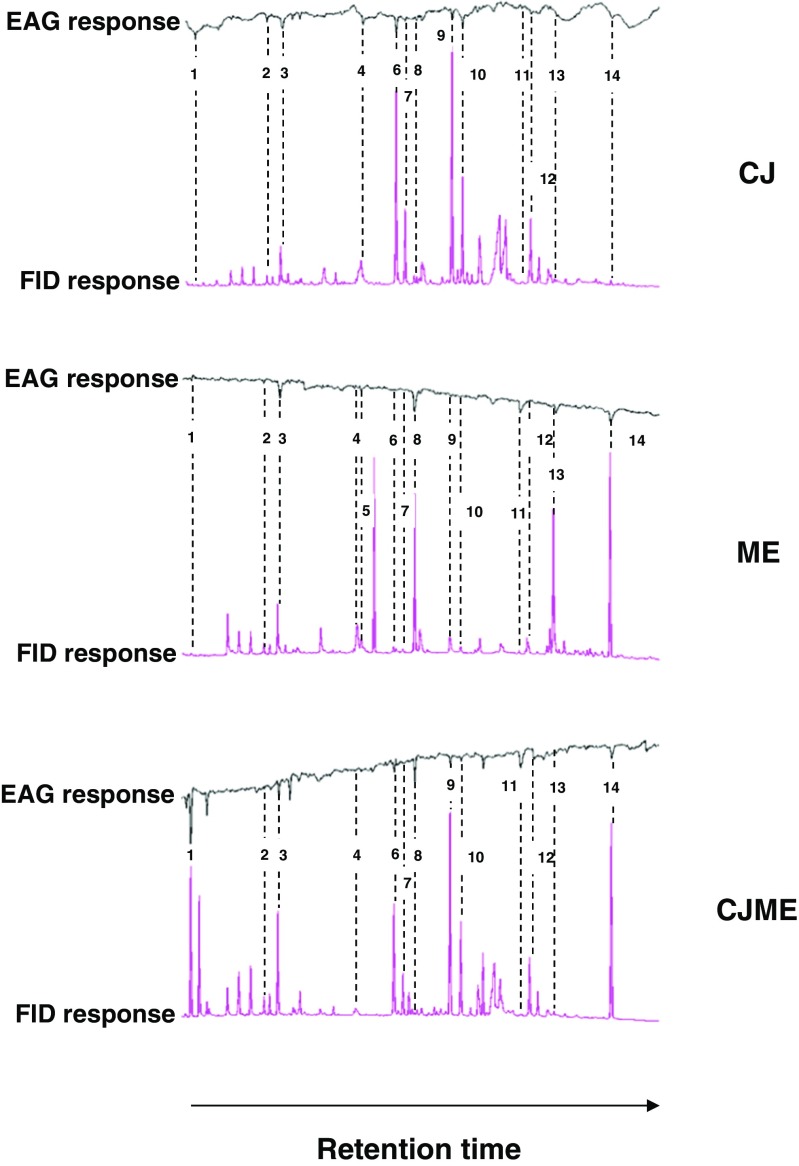
Coupled GC-EAG analysis showing antennal response of female *Macrosiphum euphorbiae* to volatile organic compound (VOC) samples collected from potato (*Solanum tuberosum* L.) plants following different treatments. *cis*-Jasmone (CJ) = CJ treatment. ME = *Macrosiphum euphorbiae*–infested. CJME = CJ treatment and then infestation with *M. euphorbiae*. For aphid treatments, each plant was infested with 100 apterous individuals. Upper trace = antennal response, lower trace = FID response. The EAG-active VOCs for *M. euphorbiae* were identified as: (1) (*E*)-2 hexenal; (2) α-pinene; (3) 6-methyl-5-hepten-2-one (MHO); (4) methyl benzoate (MeBA); (5) nonanal; (6) (*E*)-4,8-dimethyl-1,3,7-nonatriene (DMNT); (7) (*Z*)-3-hexen-1-yl butyrate; (8) methyl salicylate (MeSA); (9) decanal; (10) indole; (11) CJ; (12) α-copaene; (13) (*E*)-β-farnesene; and (14) (*E,E*)-4,8,12-trimethyl-1,3,7,11-tridecatetraene (TMTT)

Coupled GC-electroantennography (GC-EAG) analysis using alate *M. euphorbiae* showed electrophysiological activity for 14 compounds in CJ, ME, and CJME VOC samples (Fig. 2). Electrophysiological responses relating to several other peaks were demonstrated, with compounds tentatively identified by GC-MS and confirmed by GC peak enhancement as (*E*)-2 hexenal, α-pinene, 6-methyl-5-hepten-2-one (MHO), methyl benzoate (MeBA), nonanal, (*E*)-4,8-dimethyl-1,3,7-nonatriene (DMNT), (*Z*)-3-hexen-1-yl butyrate, methyl salicylate (MeSA), decanal, indole, CJ, α-copaene, (*E*)-β-farnesene, and (*E*,*E*)-4,8,12-trimethyl-1,3,7,11-tridecatetraene (TMTT).

### Multivariate Analysis of Electrophysiologically Active VOCs

**Fig. 3:**
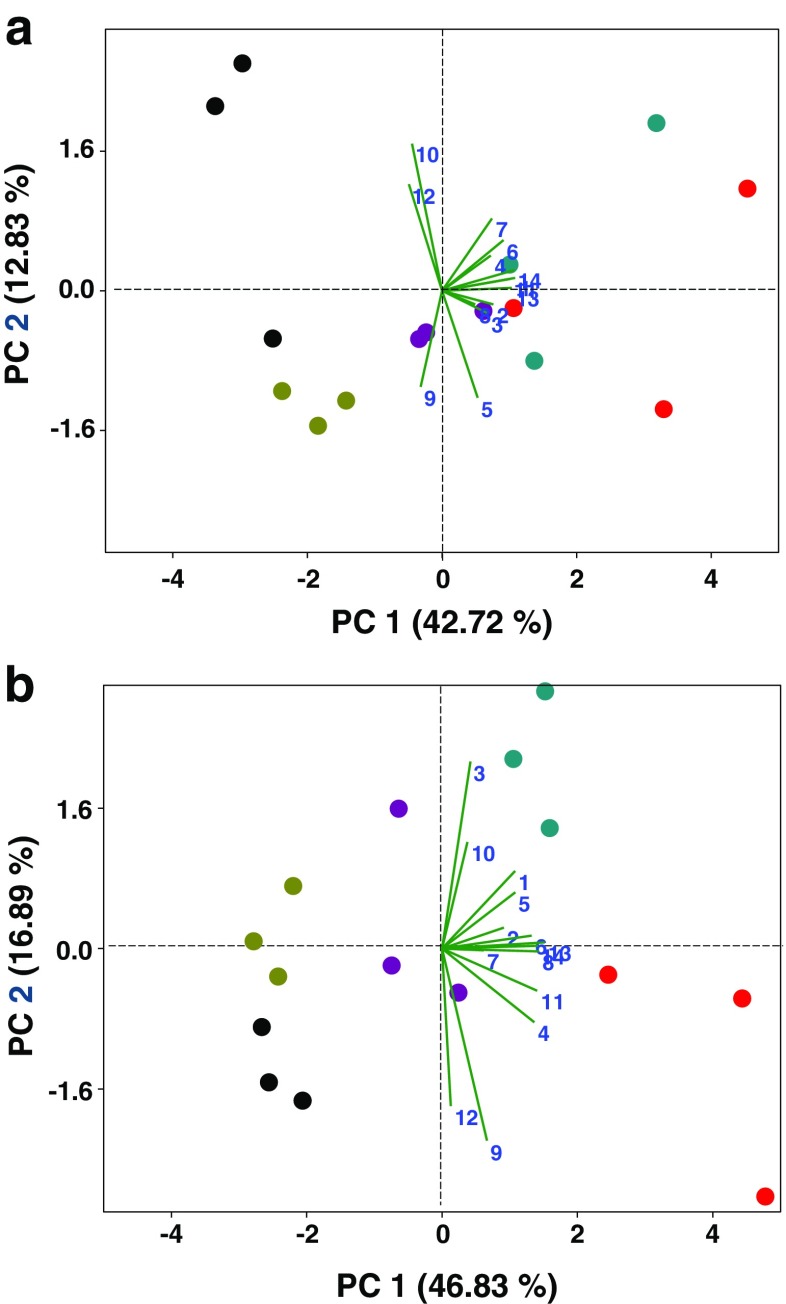
Principal Component Analysis (PCA) of the 14 EAG-active volatile organic compounds (VOCs) emitted from potato (*Solanum tuberosum*) plants, following different treatments i.e. (*black circle*) INTACT plants = neither cis-jasmone (CJ) treatment nor *Macrosiphum euphorbiae*–infested, (*violet circle*) CJ = CJ treatment, (*light blue circle*) ME = *M. euphorbiae*–infested, (*red circle*) CJME = CJ treatment and then infestation with *M. euphorbiae* and (*brown circle*) SUR = surfactant treatment. Scatter plots visualize the location of each collected sample on each PC at 48 hr (a) and 96 hr (b) with the percentage of explained variation in parentheses, whereas vectors (*green line*) visualize the loadings for each VOC. The numbers in the graphs refer to the vectors for the following compounds: (1) (*E*)-2-hexenal, (2) α-pinene, (3) 6-methyl-5-hepten-2-one, (MHO), (4) methyl benzoate (MeBA), (5) nonanal, (6) (*E*)-4,8-dimethyl-1,3,7-nonatriene (DMNT), (7) (*Z*)-3-hexen-1-yl butyrate, (8) methyl salicylate (MeSA), (9) decanal, (10) indole, (11) CJ, (12) α-copaene, (13) (*E*)-β-farnesene, (14) (*E,E*)-4,8,12-trimethyl-1,3,7,11-tridecatetraene (TMTT). For aphid treatments, each plant was infested with 100 apterous individuals

Principal component analysis (PCA) of the 14 electrophysiologically active VOCs showed that the first two components accounted for 55.55 and 63.72% of the total variation in the data at 24–48 and 72–96 hr, respectively (Fig. 3). Overall, PCA revealed that the largest separation was for VOCs of controls (i.e.,: INTACT & SUR) from VOCs of treated potato plants. Furthermore, a noticeable separation was found between samples of 24–48 hr VOCs of CJME and either ME or CJ treated samples, which was even clearer between samples of 72–96 hr VOCs. Inspection of the VOCs for the 72–96 hr period revealed greater production of many of them for the CJME treatment (Table 3), which could help explain the separation seen in the PCA (Fig. 3b). Similar results for PCA were found when the whole blend of detected VOCs was considered (Fig. A supplementary data), with the CJME treatment again becoming more differentiated from the other treatments at 72–96 hr compared to the 24–48 hr period. Analyzing the PC1 scores using one-way ANOVA, there was a significant effect of treatment (24–48 hr: *F*_4,14_ = 17.82, *P* < 0.001; 72-96 h: *F*_4,14_ = 51.21, *P* < 0.001), where PC1 accounts for 42.72 and 46.83% variance in the data for 24–48 hr and 72–96 hr, respectively (Fig. 3). A similar pattern of treatment significance on PC2 scores also was observed for the 72–96 hr collection point (*F*_4,14_ = 7.182, *P* = 0.005), which was not the case for PC2 scores at 24-48 h (*F*_4,14_ = 2.387, *P* = 0.121). The greatest loadings of PC1_48h_ were for (*E*)-2-hexenal (0.374), TMTT (0.369), (*E*)-β-farnesene (0.356), CJ (0.347) and DMNT (0.315). Likewise, major loadings of PC1_96h_ were for (*E*)-β-farnesene (0.376), MeSA (0.355), TMTT (0.352), CJ (0.348) and MeBA (0.338). This suggests that these compounds were the main VOCs contributing to PC1 and subsequently to insect behavioral responses.

### Aphid Olfactometer Bioassay

**Fig. 4:**
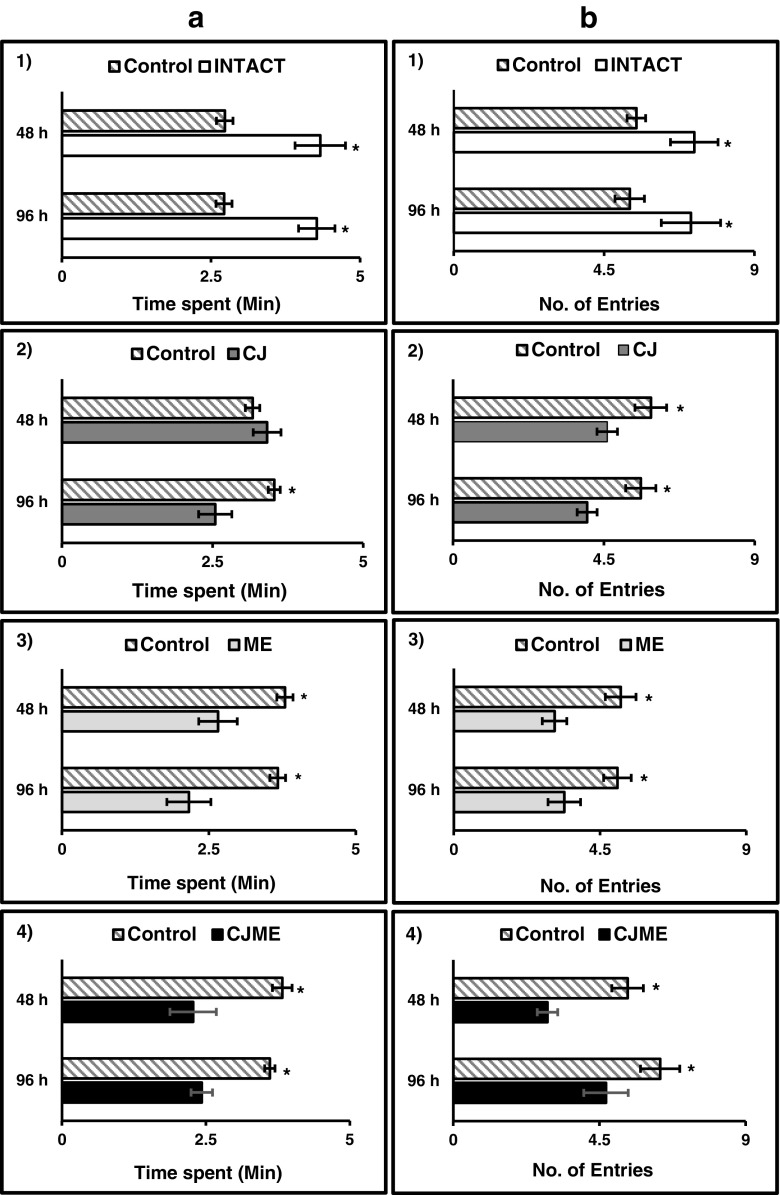
Responses of alate *Macrosiphum euphorbiae* females to potato volatile organic compounds (VOCs) in the four-arm olfactometer (one treated arm vs. three control arms). **a** Time spent = Mean time spent in arm ±SE. **b** Entries = Mean number of entries in arm ± SE. 48 h = Potato VOCs collected 24–48 hr after collections commenced, 96 hr = Potato VOCs collected 72–96 hr after collections commenced. Ten replicates were done for each assay. *Asterisks* indicate statistically significant differences (*P* < 0.05, *t*-tests)

In a first series of assays (one VOC arm vs. three solvent control arms), with significance assigned via *t*-tests, *M. euphorbiae* spent significantly more time in the presence of VOCs from INTACT plants collected during 24–48 hr (*P* = 0.011) or 72–96 hr after volatile collection was initiated (*P* = 0.003), compared to solvent controls (Fig. 4_A,1_). The same was true for the number of entries to the treated arm compared to the control arms (24–48 hr: *P* = 0.022; 96 hr: *P* = 0.017; Fig. 4_B,1_). *M. euphorbiae* did not distinguish between the VOCs of CJ*-*treated potato plants collected during 24-48 h and the solvent control (*P* = 0.435), but spent significantly less time in the presence of VOCs from CJ-treated plants collected during 72–96 hr (*P* = 0.023, Fig. 4_A,2_). Significantly fewer entries to the treated arm for *M. euphorbiae* were observed for both 24–48 hr and 72–96 hr samples collected from CJ-treated plants (24–48 hr: *P* = 0.042; 72–96 hr: *P* = 0.039; Fig. 4_B,2_). Adults of *M. euphorbiae* spent significantly less time in the presence of VOCs from *M. euphorbiae*-infested plants, either pre-treated with CJ (CJME_48h_: *P* = 0.022; CJME_96h_: *P* = 0.001, Fig. 4_A,4_) or not (ME_48h_: *P* = 0.023; ME_96h_: *P* = 0.014, Fig. 4_A,3_). Similarly, entries by *M. euphorbiae* to CJME or ME arms were notably fewer [(Fig. 4_B3,4_; CJME_48h_ (*P* = 0.001); CJME_96h_ (*P* = 0.005); ME_48h_ (*P* = 0.009); ME_96h_ (*P* < 0.001)].

**Fig. 5:**
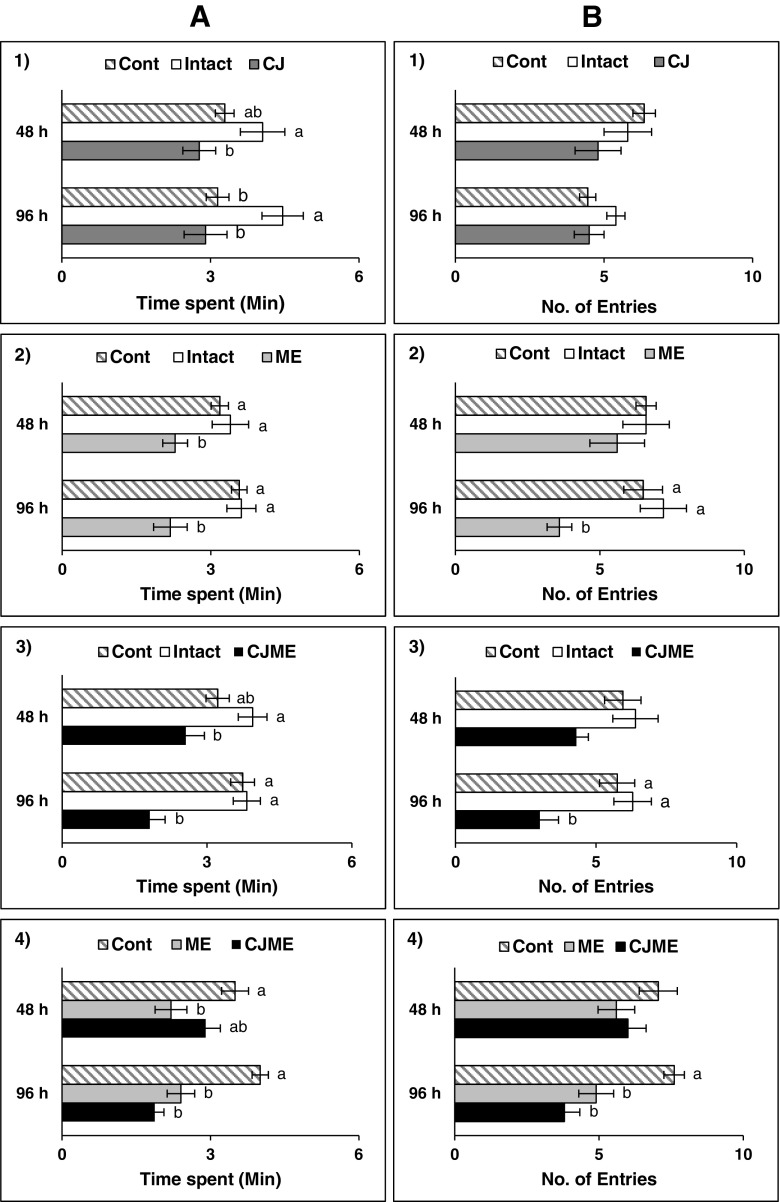
Responses of alate *Macrosiphum euphorbiae* females to potato volatile organic compounds (VOCs) in the four-arm olfactometer with two different treatment arms (Intact plants = neither *cis*-jasmone (CJ) treatment nor *M. euphorbiae*–infested, CJ = CJ treatment, ME = *M. euphorbiae*–infested and CJME = CJ treatment and then infestation with *M. euphorbiae*) vs. two control arms. **A** Time spent = Mean time spent in arm ±SE. **B** Entries = Mean number of entries in arm ±SE. 1) Control – Intact – CJ; 2) Control – Intact ME, 3) Control – Intact CJME, 4) Control – ME – CJME treatments. 48 hr = Potato VOCs collected 24–48 hr after collections commenced, 96 hr = Potato VOCs collected 72–96 hr after collections commenced. Ten replicates were done for each assay. For each assay by time point combination, means with different letters indicate statistically significant differences [*P* < 0.05; Student-Newman-Keuls method, or Dunn’s method (panel B, 2)]

In a second set of assays, the behavior of *M. euphorbiae* to two VOC treated arms vs. two solvent control arms was tested invoking *F*-tests or *H*-tests followed by the Student-Newman-Keuls method or Dunn’s method, respectively, for statistical separation of means. Overall, CJ, ME, and CJME VOCs had a repellent effect on *M. euphorbiae,* as they spent less time in olfactometer arms containing these VOCs (Figs. 5_A1–3_). *M. euphorbiae* spent significantly less time in arms containing CJ VOCs than INTACT controls (24–48 hr: *F*_2,29_ = 3.58, *P* = 0.042; 72–96 hr: *F*_2,29_ = 5.06, *P* = 0.014; Fig. 5_A1_). The same tendency was also observed for ME and CJME VOCs [ME_48h_ (*F*_2,29_ = 4.55, *P* = 0.020); ME_96h_ (*F*_2,29_ = 8.84, *P* = 0.001); CJME_48h_ (*F*_2,29_ = 4.87, *P* = 0.016); CJME_96h_ (*F*_2,29_ = 16.17, *P* = <0.001); Fig. 5_A2,3_]. Similarly, entries of *M. euphorbiae* were significantly fewer for ME_96h_ (*H* = 11.87, *P* = 0.003; Fig. 5_B2_) and CJME_96h_ (*F*_2,29_ = 7.32, *P* = 0.003; Fig. 5_B3_) arms. In contrast, *M. euphorbiae* did not distinguish between control and treated arms containing VOCs collected at 24-48 h (Fig. 5_B1,2,3_).

To elucidate which VOC blend had greater repellency for *M. euphorbiae*, a third assay was performed in which *M. euphorbiae* were given a choice of ME and CJME VOCs vs. two solvent controls (Fig. 5_4_). For VOCs collected during the 24–48 hr period, *M. euphorbiae* spent less time in the presence of ME VOCs than either CJME or control arms (*F*_2,29_ = 4.75, *P* = 0.017; Fig. 5_A4_), but in terms of entries, aphids could not distinguish between different VOCs (*F*_2,29_ = 1.35, *P* = 0.275; Fig. 5_B4_). Stronger repulsion to both treated VOC arms was observed with regards to the time spent or entries by *M. euphorbiae* females for 72–96 hr entrainments (time spent: *F*_2,29_ = 26.52, *P* <0.001; entries:*F*_2,29_ = 14.76, *P* < 0.001; Fig. 5_A4_, _B4_).

## Discussion

Aphids are major pests of agricultural crops worldwide because of direct damage to crops and as vectors of plant viruses (Blackman and Eastop 2006; Saguez et al. 2013). To locate host plants, aphids employ sophisticated behavioral mechanisms. An understanding of these consecutive events may lead to improved management strategies (Powell et al. 2006). Plants have evolved highly effective defense mechanisms to resist being consumed by herbivorous insects in general (Mithöfer and Boland 2012) and aphids in particular (Züst and Agrawal 2016), with these inducible defensive mechanisms being regulated mainly by jasmonic acid (JA), which is the major hormone associated with insect/herbivore responses in terms of the production and release of VOCs (Howe and Jander 2008). Such responses also can be induced by the application of plant elicitors (Smith et al. 2009; Sobhy et al. 2012), which is advantageous in the elucidation of plant/aphid interactions. This study has determined the impact of the natural plant stress signal and elicitor *cis*-jasmone (CJ) on the interaction between potatoes and the potato aphid *M. euphorbiae*. GC and coupled GC-MS data showed that treating potato plants with CJ increased the total emission of VOCs, with emission increasingly elevated until after the 72–96 hr period. CJ treatment not only increased the number of emitted VOCs but also enhanced the levels of components such as DMNT, MeSA, (*E*)-β-farnesene and TMTT, which are key VOCs in plant/insect interactions. CJ treatment is known to increase the emission of these VOCs in other crop plants, for example, DMNT, (*E*)-ocimene, MeSA, and TMTT in soybean and cotton (Hegde et al. 2012; Moraes et al. 2009). Using potato, we showed that CJ treatment induces the release of a similar profile of defense VOCs as released by *M. euphorbiae* herbivory. This relates to other findings from Hegde et al. (2012), who showed that CJ can induce the production of *A. gossypii*-induced VOCs from cotton plants. Furthermore, Dewhirst et al. (2012) confirmed that collected VOCs from CJ-treated sweet pepper plants are quantifiably different compared to those from untreated plants.

The factorial analysis of our data in this study revealed that there was a synergistic interaction between CJ treatment and aphid herbivory in the emission of certain VOCs. Similar findings were reported by Sobhy et al. (2015), where qualitative and quantitative differences in VOC production were observed from herbivore-damaged cotton upon treatment with plant strengtheners. For CJ-treated potato plants, CJ application may lead to a memory (priming) effect of VOC production, which can be attributed to subsequent changes in the transcriptome (Matthes et al. 2010, 2011; Moraes et al. 2008). Additionally, Menzel et al. (2014) reported that a low dose of JA results in a synergistic effect on gene transcription, which thereby increases the emission of VOCs involved in indirect defense after herbivore infestation.

Coupled GC-EAG analysis performed with *M. euphorbiae* alates showed that 14 VOCs were electrophysiologically active on this insect species in both aphid and CJ treatments. We then showed repellence of *M. euphorbiae* by the VOCs collected from CJ, aphid, and CJ/aphid-treated potato plants. Given that VOCs collected from CJ-treated plants during 72–96 hr were most repellent, this reinforces our conclusion that changes in VOC composition elicit anti-herbivore activity or plant antixenosis to *M. euphorbiae* alate females. Webster et al. (2010) found that (*E*)-2-hexenal, MeSA, decanal, (*E*)-β-farnesene and TMTT elicited negative behavioral responses from *Aphis fabae*. Similar findings were reported by Hegde et al. (2011), who showed that MeSA and TMTT were repellent to *A. gossypii*. Many reports emphasize that emission of MeSA increases following aphid attack in a number of plant/aphid systems (Sasso et al. 2007; Zhu and Park 2005). Such increases are used by aphids to avoid overexploited hosts (Hardie et al. 1994). Congruent to our findings, Gosset et al. (2009) found that (*E*)-2-hexenal, α-pinene, nonanal and (*E*)-β-farnesene are released at high rates by potato plants following attack by *M. persicae*. It has been suggested that small amounts of (*E*)-2-hexenal are also released from intact potato plants during the middle of the day, but that huge amounts of (*E*)-2-hexenal are released 0–5 min after plant damage (Agelopoulos et al. 1999). Although the release of MHO from tomato plants is not increased by the presence of *M. euphorbiae*, the compound has been shown to possess biological activity (Sasso et al. 2007). Pickett et al. (2007) reported that certain elite cultivars of winter wheat increase MHO emission, which is highly active in reducing aphid colonization, following aphid attack. In addition, da Costa et al. (2010) reported that MHO is an important host-derived semiochemical for *A. gossypii*. Another active compound for *M. euphorbiae* is DMNT, which has been reported as being repellent to *A. gossypii* upon treatment of cotton with CJ (Hegde et al. 2012). Our results show that methyl benzoate (MeBA) is also an electrophysiologically active VOC for *M. euphorbiae,* which equates to findings by Staudt et al. (2010), who showed that MeBA is emitted by aphid-infested plants. *M. euphorbiae* responded to the sesquiterpene α-copaene, which is emitted in a notable amount from apple trees infested by the rosy apple aphid, *Dysaphis plantaginea* (Stewart-Jones and Poppy 2006). Both (*Z*)-3-hexen-1-yl butyrate and indole have been reported as induced VOCs from potato plants infested by Colorado potato beetle (*Leptinotarsa decemlineata*) after 2 and 20 hr feeding (Bolter et al. 1997). Using another solanaceous plant, Dewhirst et al. (2012) found that induced sweet pepper plants emitted elevated levels of (*Z*)-3-hexen-1-yl butyrate compared to control plants. Our findings show that CJ is an aphid-induced VOC from potatoes, which is consistent with earlier findings by Birkett et al. (2000), who showed that CJ was not only electrophysiologically active for the lettuce aphid, *Nasonovia ribis-nigri*, but also had a repellent effect. Using principal component analysis (PCA) to analyze the VOC data, (*E*)-β-farnesene, (*E*)-2-hexenal, TMTT, MeSA and CJ had the greatest loadings for PC1 (which appeared to be associated with a separation of the treatments) from both the 24–48 hr and 72–96 hr air entrainment periods (Fig. 3), thus suggesting that production of these compounds correlates most strongly with aphid repellence.

Several studies have addressed the importance of these compounds in plant/aphid interactions (Bruce and Pickett 2011; Dewhirst and Pickett 2009). Brunissen et al. (2010) found that treating potato plants with methyl jasmonate decreased its attractiveness to *M. euphorbiae*. Results here (Fig. 5) show that CJME_96h_ gave release of VOCs most repellent to *M. euphorbiae*, which may have arisen as a consequence of phytotoxic effects by this treatment. Particularly promising is the finding of inducible responses following CJ application in unrelated plant species of economic importance. Thus, treatment of cereal crops with CJ reduces aphid infestation (Bruce et al. 2003a, b; Delaney et al. 2013) and analogous impacts have been reported for many dicot crops (Dewhirst et al. 2012; Hegde et al. 2012; Moraes et al. 2009). The general inducible effect of CJ on various crops would be highly beneficial when using a formulation of it in field applications, for example on mixed arable farms. However, due to the fact that plant response to these elicitors is genotype-specific (Bruce 2014), more experimentation is required before CJ can be used in a practical way by growers for crop protection.

In summary, the results of this study provide evidence that CJ elicits potato defense similar to that observed for other important staple crops (Hegde et al. 2012; Moraes et al. 2008). Such general patterns of activity suggest the possibility of enhancing defense in crop plants *via* the development of plant defense activators (Pickett et al. 2014). Given the mounting interest in manipulating plant semiochemicals with the use of plant elicitors as a new tactic for protecting crops against insect pests (Sobhy et al. 2014; Stenberg et al. 2015), we suggest that CJ could provide such a benign tool. However, further studies are required to determine the underlying mechanisms that alter VOC emission, which subsequently manipulates aphid behavior. Given that the potato is of global importance and contributes to the economy of many developing countries, optimization of yield per cultivated area requires the application of appropriate and ecologically safe agricultural technologies. Plant activators such as CJ have the potential to be adopted as a strategy to enhance plant defense against herbivorous insects.

## Electronic supplementary material

Fig. 1 (supplementary data). Principal Component Analysis (PCA) biplots of the 29 detected volatile organic compounds (VOCs) emitted from potato (*Solanum tuberosum*) plants, following different treatments i.e. (●) INTACT plants = neither *cis*-jasmone (CJ) treatment nor *Macrosiphum euphorbiae*–infested, (●) CJ = *cis*-jasmone treatment, (●) ME = *M. euphorbiae*–infested, (●) CJME = CJ treatment and then infestation with *M. euphorbiae* and (●) SUR = surfactant treatment. For aphid treatments, each plant was infested with 100 apterous individuals. Scatter plots visualize the pattern of emitted VOCs collected at 48 hr (A) and 96 hr (B) after collections commenced. (PPTX 41 kb)

Fig. 2 (PPTX 111 kb)
