# Supplementary figures and images for: cis-Jasmone Elicits Aphid-Induced Stress Signalling in Potatoes
Source: J Chem Ecol. 2017 Jan 27;43(1):39–52. doi: 10.1007/s10886-016-0805-9 (PMC5331074; doi:10.1007/s10886-016-0805-9)

## Slide 1
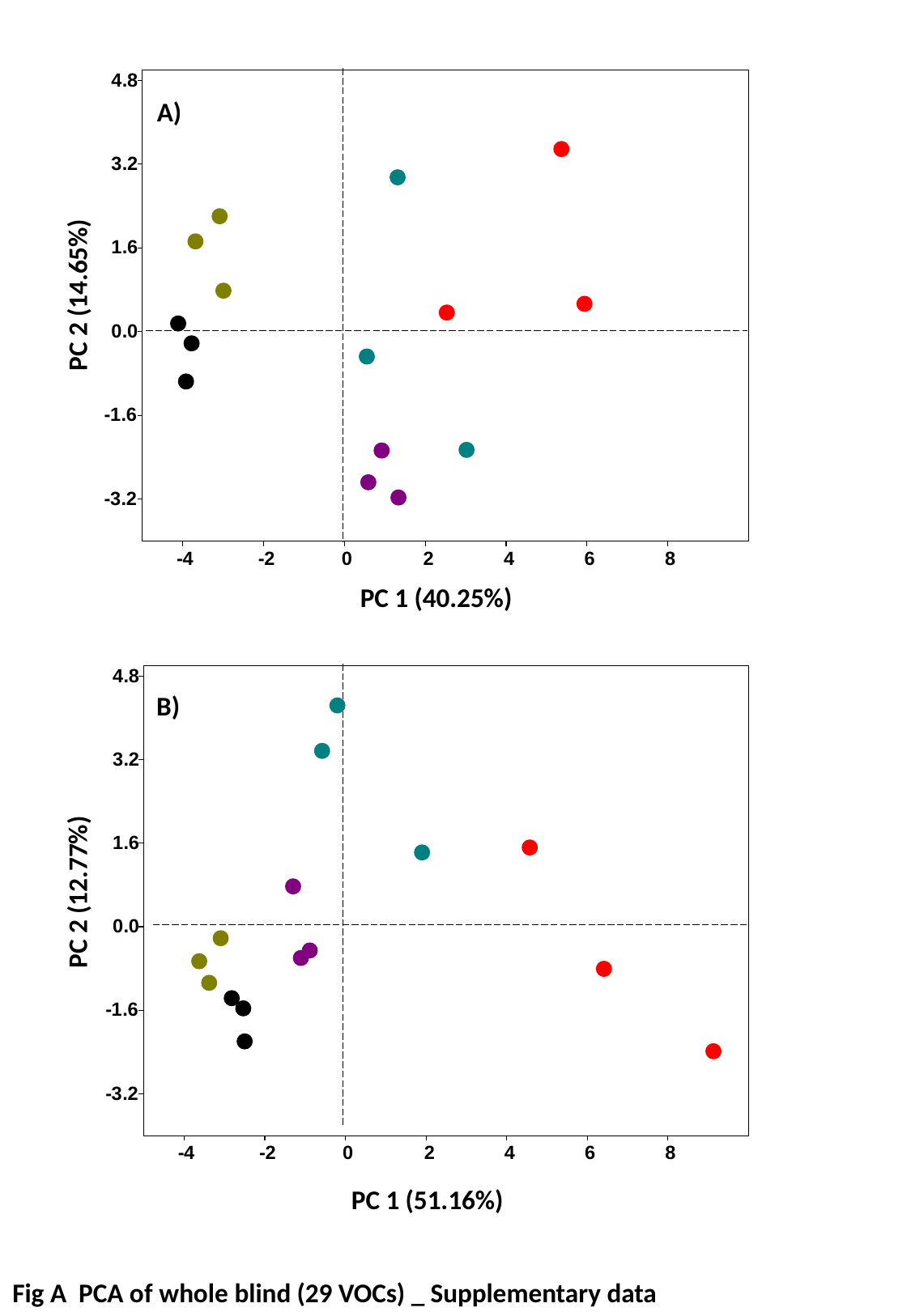

A)
PC 2 (14.65%)
PC 1 (40.25%)
B)
PC 2 (12.77%)
PC 1 (51.16%)
Fig A PCA of whole blind (29 VOCs) _ Supplementary data

Supplement: Supplementary file 1 — (supplementary data). Principal Component Analysis (PCA) biplots of the 29 detected volatile organic compounds (VOCs) emitted from potato (Solanum tuberosum) plants, following different treatments i.e. (●) INTACT plants = neither cis-jasmone (CJ) treatment nor Macrosiphum euphorbiae–infested, (●) CJ = cis-jasmone treatment, (●) ME = M. euphorbiae–infested, (●) CJME = CJ treatment and then infestation with M. euphorbiae and (●) SUR = surfactant treatment. For aphid treatments, each plant was infested with 100 apterous individuals. Scatter plots visualize the pattern of emitted VOCs collected at 48 hr (A) and 96 hr (B) after collections commenced. (PPTX 41 kb) [file 10886_2016_805_MOESM1_ESM.pptx]

## Slide 1
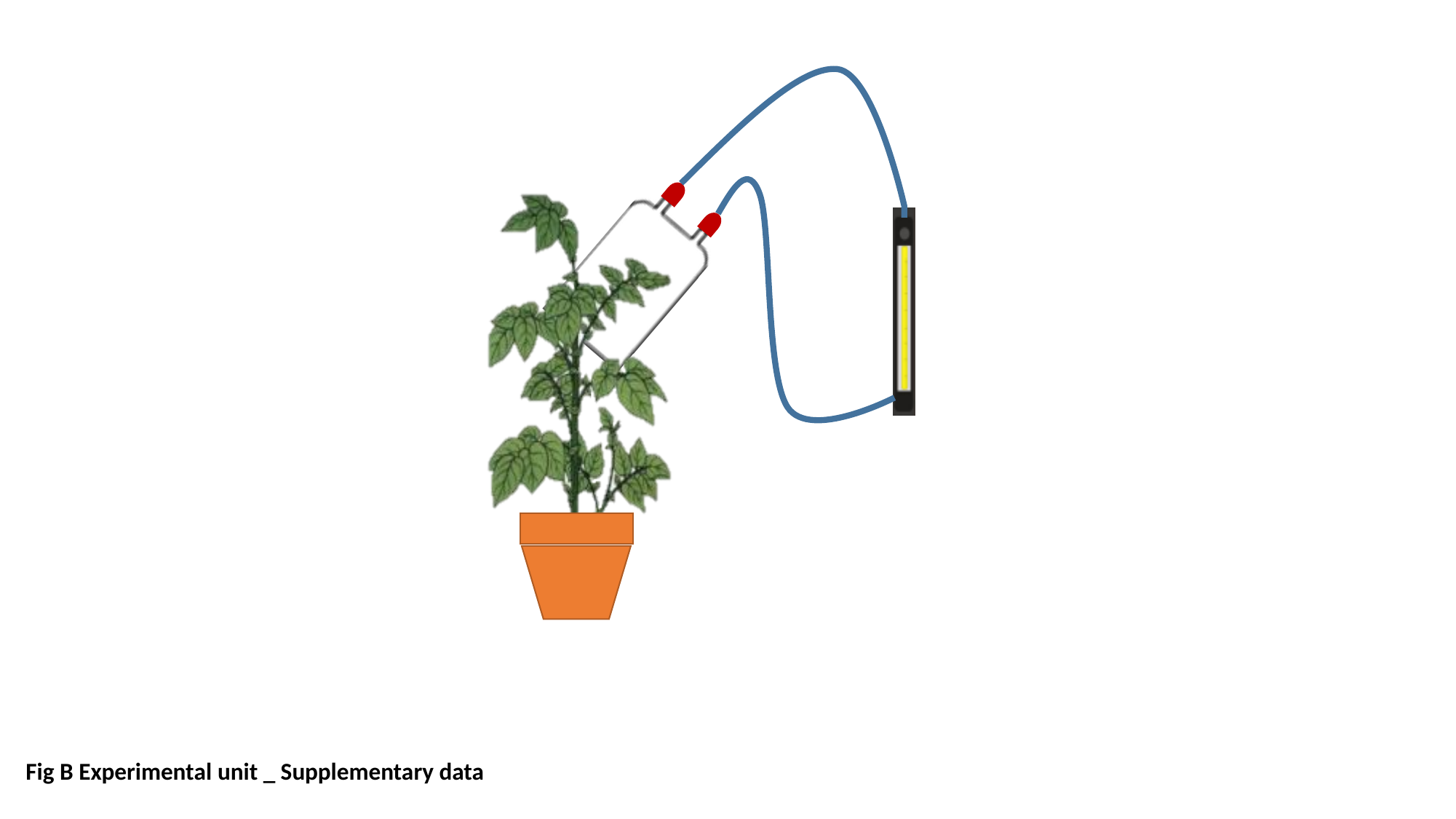

Fig B Experimental unit _ Supplementary data

Supplement: Supplementary file 2 — (PPTX 111 kb) [file 10886_2016_805_MOESM2_ESM.pptx]
